# Defective ALK5 signaling in the neural crest leads to increased postmigratory neural crest cell apoptosis and severe outflow tract defects

**DOI:** 10.1186/1471-213X-6-51

**Published:** 2006-11-01

**Authors:** Jikui Wang, Andre Nagy, Jonas Larsson, Marek Dudas, Henry M Sucov, Vesa Kaartinen

**Affiliations:** 1 Developmental Biology Program, The Saban Research Institute of Childrens Hospital Los Angeles, Departments of Pathology and Surgery, Keck School of Medicine, University of Southern California, Los Angeles, CA 90027, USA; 2 Molecular Medicine and Gene Therapy, Institute of Laboratory Medicine and Department of Medicine, Lund University Hospital, 221 00 Lund, Sweden; 3 Institute for Genetic Medicine, Keck School of Medicine University of Southern California, Los Angeles, CA 90033, USA

## Abstract

**Background:**

Congenital cardiovascular diseases are the most common form of birth defects in humans. A substantial portion of these defects has been associated with inappropriate induction, migration, differentiation and patterning of pluripotent cardiac neural crest stem cells. While TGF-β-superfamily signaling has been strongly implicated in neural crest cell development, the detailed molecular signaling mechanisms *in vivo *are still poorly understood.

**Results:**

We deleted the TGF-β type I receptor *Alk5 *specifically in the mouse neural crest cell lineage. Failure in signaling via ALK5 leads to severe cardiovascular and pharyngeal defects, including inappropriate remodeling of pharyngeal arch arteries, abnormal aortic sac development, failure in pharyngeal organ migration and persistent truncus arteriosus. While ALK5 is not required for neural crest cell migration, our results demonstrate that it plays an important role in the survival of post-migratory cardiac neural crest cells.

**Conclusion:**

Our results demonstrate that ALK5-mediated signaling in neural crest cells plays an essential cell-autonomous role in the pharyngeal and cardiac outflow tract development.

## Background

A considerable percentage of cardiac birth defects is caused by a failure in normal migration, differentiation or patterning of the cardiac neural crest (CNC). This subset of pluripotent neural crest stem cells forms in the dorsal aspect of the neural tube at the level of the mid-otic placode to the third somite [1]. Subsequently cardiac neural crest cells (CNCCs) delaminate, undergo a phenotypic transformation from an epithelial to mesenchymal cell type, and migrate latero-ventrally into the 3^rd^, 4^th ^and 6^th ^pharyngeal arch arteries (PAAs), where they contribute to the formation of the smooth muscle cell layer of endothelial structures derived from the aortic arch arteries [1-3]. A subset of CNCCs continues to migrate deeper into the aortic sac to form the aortico-pulmonary septum; a vital structure, which separates the pulmonary trunk from the aorta [4].

An indispensable role of CNCCs in the development of the cardiac outflow tract was originally demonstrated by pioneering studies of Kirby and coworkers [1], who showed that ablation of the CNC in the chick led to severe outflow tract (OFT) defects including persistent truncus arteriosus (PTA), mispatterning of the great vessels and outflow tract mal-alignments [5]. Early migratory CNCCs have been shown to retain a considerable degree of plasticity and their fate is largely controlled by instructional signals from local environments into which NCCs migrate [6]. Several recent studies have indicated that members of the TGF-β superfamily, i.e., TGF-βs and BMPs are likely candidates to provide some of these signals. Mice deficient in TGF-β2 display fourth aortic arch artery defects [7], while neural crest cell specific abrogation of TGF-β type II receptor (*Tgfbr2*) results in interruption of the aortic arch and PTA [8,9]. BMPs 6 and -7 are required for proper formation of the outflow tract cushions [10], while BMP type II receptor is needed for proper development of the conotruncal ridges [11]. Moreover, neural crest-specific deletion of the BMP type I receptors *Alk2 *and *Alk3 *has been shown to lead to defective aortico-pulmonary septation, among other cardiac defects [12,13].

TGF-β subfamily ligands signal via a receptor complex composed of two type II receptors and two type I receptors [14,15]. Ligand binding leads to phosphorylation and activation of type I receptors, which, in turn, phosphorylate and activate a specific set of downstream signaling molecules called Smads. In general terms, TGF-βs bind to the TGF-β type II receptor (TGFβRII) and TGF-β type I receptor (ALK5) activating TGF-β Smads (2 and 3), while BMPs bind to the BMP type II receptor and type I receptors ALK2, -3, or 6, activating BMP Smads (1, 5 and 8). However, it is likely that these signaling interactions are more complex *in vivo*, possibly allowing formation of heterotetrameric complexes composed of different type II and type I receptors [16]. In addition, recent studies have identified novel TGF-β-related ligands, which can bind to entire different combinations of receptors. For instance, growth and differentiation factors (GDFs) 8 and 9 can bind to Activin type II receptor and ALK5 to activate TGF-β Smads [17,18]. Therefore, we hypothesized that deletion of *Alk5 *in a specific cell lineage should reveal phenotypes which cannot be seen in comparable mutants lacking *Tgfbr2*. Indeed, we recently showed that neural crest cell specific *Alk5 *mutants display a unique spectrum of craniofacial developmental defects, e.g., cleft snout and severe mandibular hypoplasia [19]; these phenotypes were not seen in corresponding *Tgfbr2 *mutants [20]. To determine, whether ALK5 would also mediate unique non-redundant signaling events in cardiac neural crest cells, we focused on cardiac and pharyngeal phenotypes of mouse embryos lacking *Alk5 *specifically in neural crest cells. We discovered that in *Alk5/Wnt1-Cre *mutants, pharyngeal organs (thymus and parathyroid) fail to migrate appropriately. Moreover, the mutant embryos display severe aortic sac and pharyngeal arch artery defects, and failed aortico-pulmonary septation leading to PTA. Our data further suggest that at least some of these abnormal detected phenotypes result from a dramatic increase in apoptosis of postmigratory cardiac neural crest cells. These phenotypes differ remarkably from those seen in corresponding *Tgfbr2 *mutants, suggesting that ALK5 mediates a wider spectrum of signaling events than its classical binding partner TGFβRII in cardiac neural crest cells during cardiac and pharyngeal development.

## Results

### Persistent truncus arteriosus and abnormal large vessels in mice lacking *Alk5 *in cardiac NCCs

To inactivate *Alk5 *in cardiac NCCs, mice homozygous for the *floxed Alk5 *allele (*Alk5*^*Flox*/*Flox*^) [21] were crossed with transgenic *Wnt1-Cre *mice [22], which were also heterozygous for the *Alk5 *knockout allele (*Alk5*^*KO*^) allele. The resulting mice heterozygous for the *Alk5*^*Flox *^and *Alk5*^*KO *^alleles, which also carried the *Wnt1-Cre *transgene, had the *Alk5 *gene specifically inactivated in NCCs (herein termed *Alk5/Wnt1-Cre*), while the littermates with remaining allelic combinations were phenotypically normal and served as controls (*Alk5*^*Flox*/+^*, Alk5*^*KO*/+^*; Wnt1-Cre*). When embryos were harvested during the last day of gestation, an expected number (25%) of *Alk5/Wnt1-Cre *mutants were recovered. However, all mutant offspring died either during the birth or during the first post-natal hours.

**Figure 1:**
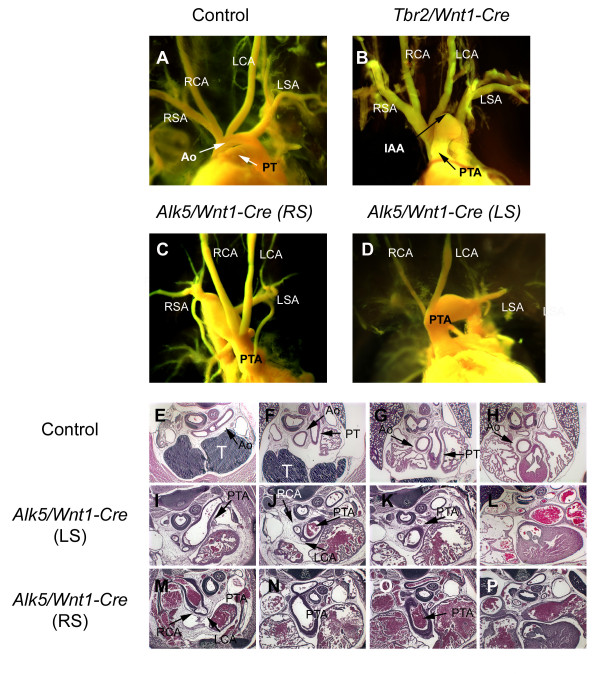
**Abrogation of *Alk5 *in neural crest cells leads to persistent truncus arteriosus type A2**. A-D, Casting-dye analysis of OFT morphogenesis at E17.0. Control (A), *Tgfbr2/Wnt1-Cre *mutant [8] (B) demonstrating the PTA type A4 (= truncus arteriosus with interrupted aortic arch [30]) and *Alk5/Wnt1-Cre *mutants demonstrating the right-sided (C) and left-sided (D) arches of the truncus. E-P, Histological cross-sections on four different levels (rostral to caudal) at E17.0. In a control (E-H), the ascending aorta (Ao) and pulmonary trunk (PT) are separated by the conotruncal septum. In *Alk5/Wnt1-Cre *mutants (I-P) the conotruncal septum fails to form, and either left-sided (I-L) or right-sided (M-P) aortic arch can be seen. Aberrant branching of carotid arteries from the truncus has been illustrated by black arrows (J and M). Ao, aorta; PT, pulmonary trunk; RSA, right subclavian artery; RCA, right carotid artery; LCA; left carotid artery; LSA, left subclavian artery; IAA, interrupted aortic arch; PTA, persistent truncus arteriosus.

To determine, if ALK5-mediated TGF-β-signaling had a role in development of the OFT and large vessels of the aortic arch, we performed casting dye experiments on E17 embryos (Fig. 1A–D). In wild-type embryos (Fig. 1A), the aorta was clearly separated from the pulmonary trunk, and the right brachiocephalic, left carotid and left subclavian arteries branched directly off the aortic arch. In contrast, *Alk5/Wnt1Cre *mutant embryos consistently displayed a single prominent arterial trunk (Fig. 1C–D), while corresponding *Tgfbr2 *mutant embryos (Fig. 1B) displayed interrupted aortic arch, as reported earlier [8]. Approximately 40% of the *Alk5 *mutants had a right-sided outflow tract, with the retroesophageal arch connecting to the descending aorta and to the left subclavian artery. The carotid arteries originated either from a common bud located in the ventral side of the ascending arch, or from separate adjacent sites, as verified by serial sectioning (Fig. 1M–P). The remaining mutants displayed a left-sided aortic arch, where the right carotid arteries originated from the right lateral aspect of the ascending trunk, while the left carotid arteries budded from the ventral or right ventral aspects of the trunk (Fig. 1I–L). Both right and left subclavian arteries consistently originated from the descending part of the aortic arch. Similarly, in all mutants both left and right pulmonary arteries always branched out from the common arterial trunk. To conclude, *Alk5/Wnt1-Cre *mutants consistently displayed PTA, which differed significantly from the characteristic interrupted aortic arch phenotype seen in *Tgfbr2/Wnt1-Cre *mutants [8,9].

### Abnormal patterning of the pharyngeal arch arteries and aortic sac in *Alk5*/*Wnt1Cre *mutants

**Figure 2:**
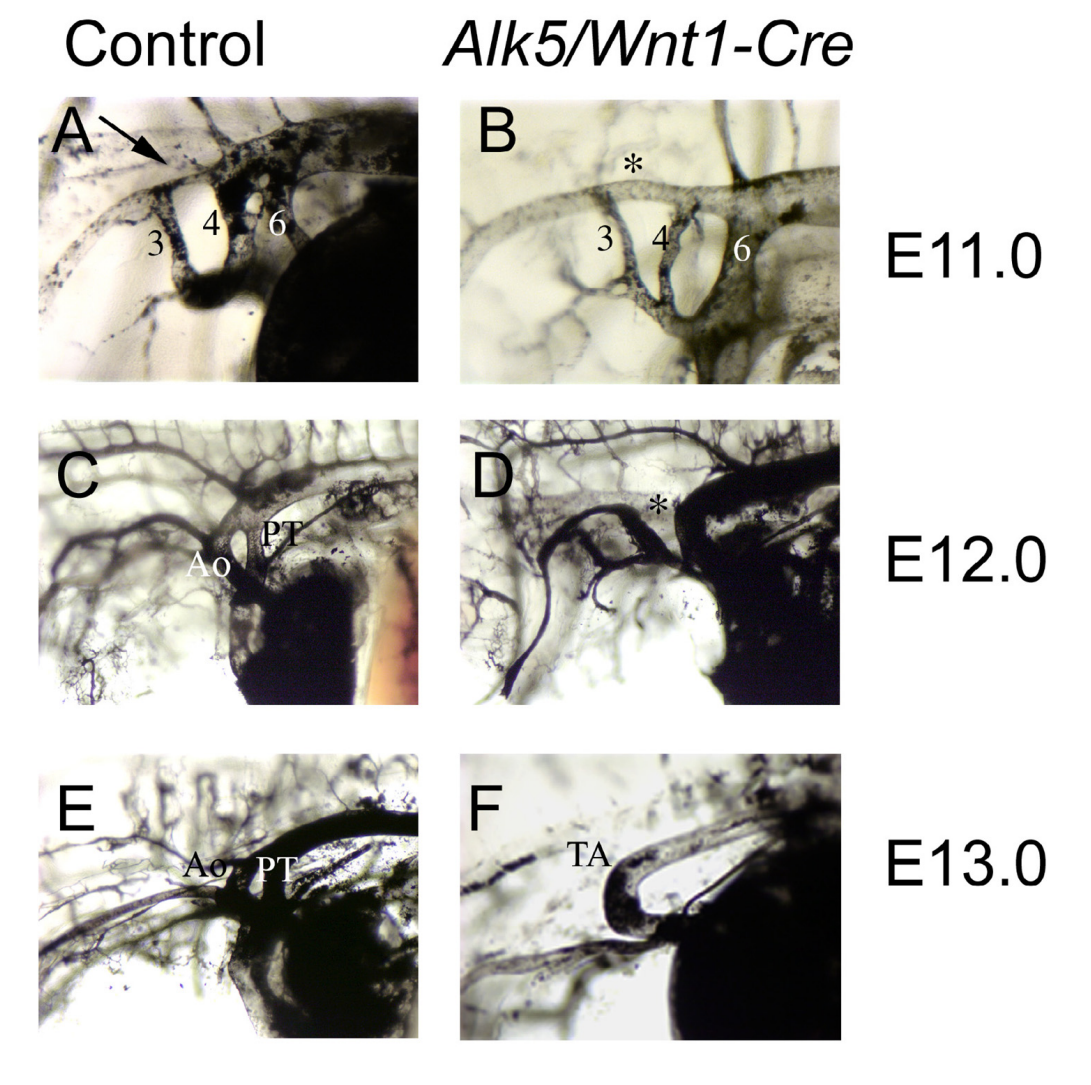
**Abnormal patterning of the PAAs in *Alk5/Wnt1-Cre *mutants**. Left lateral view after intracardiac ink injections to visualize the developing PAAs at E11.0 (A,B), E12.0 (C, D) and E13.0 (E, F) in controls (A, C, E) and *Alk5/Wnt1-Cre *mutants (B, D, F). Arrow in A points to the regressing carotid duct. Asterisk in B depicts the corresponding structure in the mutant with no signs of regression. Asterisk in D illustrates the aberrant regression of the dorsal aorta between the 4^th ^and 6^th ^PAAs. PT, pulmonary trunk; Ao, Aorta; TA, truncus arteriosus.

During cardiovascular development, the PAAs undergo a complex set of sequential asymmetric remodeling steps resulting in the left-sided aortic arch. To determine, whether ALK5-mediated signaling was involved in remodeling of PAAs, we performed intracardiac India ink injections at different developmental stages. While at E10, *Alk5/Wnt1-Cre *mutants did not show obvious differences in the PAAs, abnormal remodeling became obvious in mutants a day later at E11 (Fig. 2). The controls displayed the well-formed 3^rd^, 4^th ^and 6^th ^PAAs. Moreover, the carotid duct (the dorsal aorta between the 3^rd ^and 4^th ^PAAs) was already regressing as demonstrated by the reduced size (Fig. 2A). In *Alk5/Wnt1-Cre *mutants, the 3^rd ^and 4^th ^pairs of PAAs were bilaterally hypoplastic, whereas the 6^th ^pair of PAAs was notably hyperplastic (Fig. 2B). Furthermore, the carotid duct was remarkably large, when compared to controls. While the controls displayed an interruption of the carotid duct at E12 and E13 as expected (Fig. 2C), the mutants demonstrated an uncharacteristic break of the dorsal aorta between the 4^th ^and 6^th ^pairs of PAAs (Fig. 2D).

**Figure 3:**
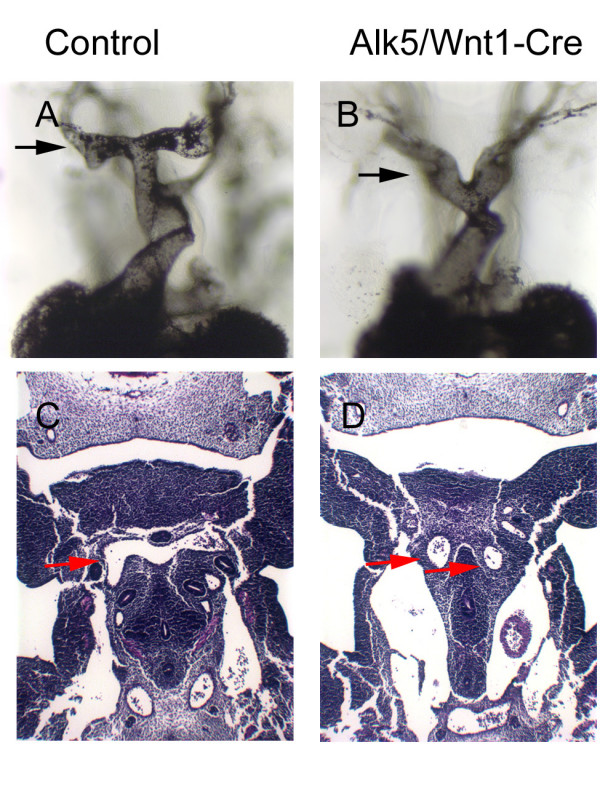
**Abnormal Aortic Sac in *Alk5/Wnt1-Cre *mutants**. *Alk5/Wnt1-Cre *mutants (B, D) fail to form the typical T-shaped structure of the aortic sac seen in controls at E11.5. (A, C). A-B, frontal image of ink-injected embryos; C-D, frontal sections (H&E staining). Arrows in A and B point to the level of section shown in C and D (red arrows in C and D point to the aortic sac of the control and mutant, respectively).

Around E11.5, the aortic sac normally forms a distinctive T-shaped structure, as seen in frontal sections of the control sample in Fig. 3(A,C). Subsequently, the right horn of this structure transforms into the prospective brachiocephalic artery, while the left horn together with the left 4^th ^PAA gives rise to the definitive aortic arch [23]. In *Alk5/Wnt1-Cre *mutants, the T-shaped aortic sac failed to form (Fig. 3B,D). Instead, the truncus bifurcated to a left and right arm, which further branched to the PAAs, particularly to the predominant pair of 6^th ^PAAs (Fig. 3B,D). The observed phenotype is consistent with the absence or severe hypoplasia of structures derived from the aortic sac in late stage embryos (E17), e.g., the missing brachiocephalic artery and severe shortening of the ascending truncus as shown in the Figure 1.

### Cardiac NCCs deficient in *Alk5 *can populate the outflow tract

**Figure 4:**
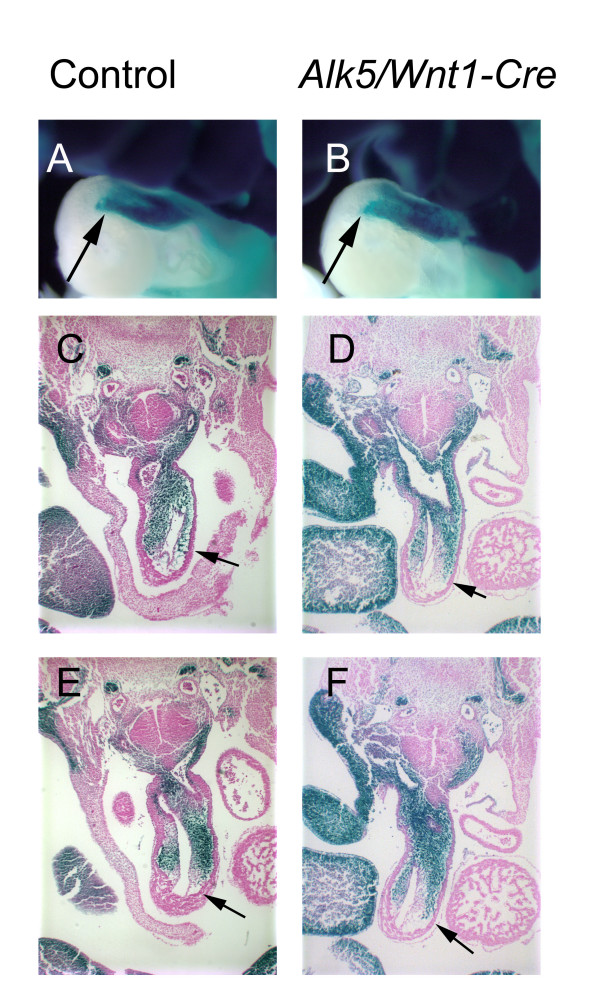
**Normal cardiac NCC migration in *Alk5/Wnt1-Cre *mutants**. The OFT of controls (A, C, E) and *Alk5/Wnt1-Cre *mutants (B, D, F) display similar staining patterns when analyzed using the *R26R *lineage tracing assay at E11.0. A-B, whole mount staining (left lateral image); C-F, transverse sections on the level of the 4^th ^(C, D) and 6^th ^(E, F) PAAs. Arrows (A-F) point to the most proximal location staining positive for the β-galactosidase activity.

Next we used the *R26R *lineage-tracing assay to determine whether CNCCs could appropriately populate the outflow tract region. Briefly, *Alk5*^*Flox*/*Flox *^mice were crossed with the *ROSA26 Cre *reporter mice, and subsequently *Alk5*^*Flox*/*Flox*^;*R26R*(+/+) females were crossed with *Alk5*^*KO*/*WT*^*;Wnt1-Cre *males. The resulting embryos had the NC-lineage permanently labeled with β-galactosidase expression, and displayed identical phenotypes to those obtained without the *R26R *reporter. Staining of embryos for β-galactosidase at E8-E11 did not reveal detectable differences in NCC migration between mutants and controls (data not shown). Similarly, serial transverse sectioning of whole mount embryos (E10-E12) and subsequent analysis of positively stained cells in the OFT region demonstrated that CNCCs deficient in *Alk5 *were capable of populating the PAAs, aortic sac and conotruncal ridges at a level comparable to that of controls (Fig 4). To conclude, the observed phenotypes in *Alk5/Wnt1-Cre *mutants were certainly not due to defective migration of CNCCs to the pharyngeal and outflow tract regions.

### Aortic sac and aortico-pulmonary septal defects in *Alk5*/*Wnt1Cre *mutant embryos

**Figure 5:**
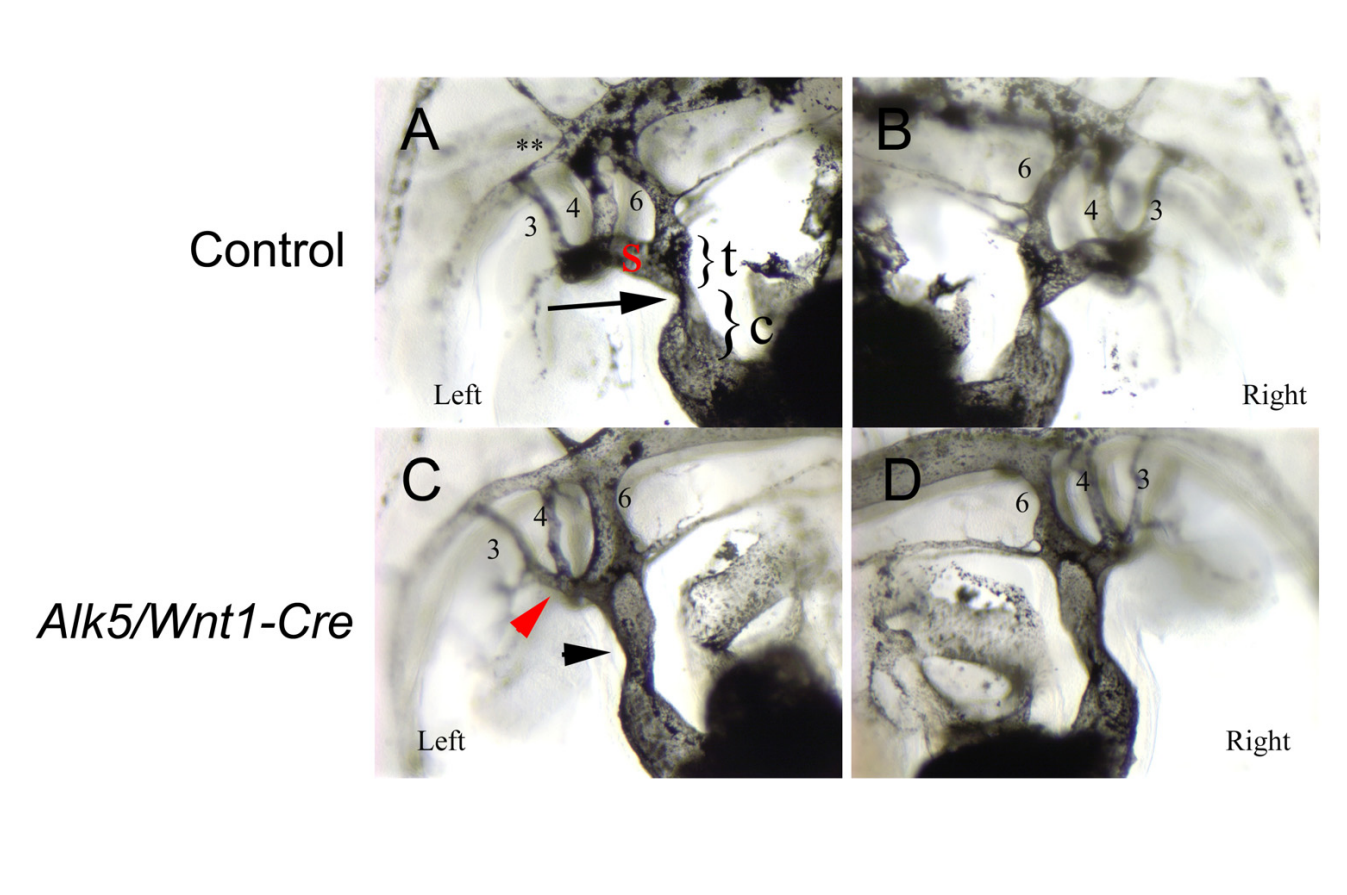
**The truncal OFT fails to rotate in *Alk5/Wnt1-Cre *mutants**. Left (A, C) and right (B, D) lateral images of ink-injected control (A-B) and mutant (C-D) embryos at E11.5 demonstrate the abnormally straight OFT in mutants lacking the typical conotruncal transition (black arrow in A vs. black arrowhead in C) seen in control. Red arrowhead (C) points to the abnormally shaped aortic sac. Red "s", aortic sac; t, truncus; c, conus.

**Figure 6:**
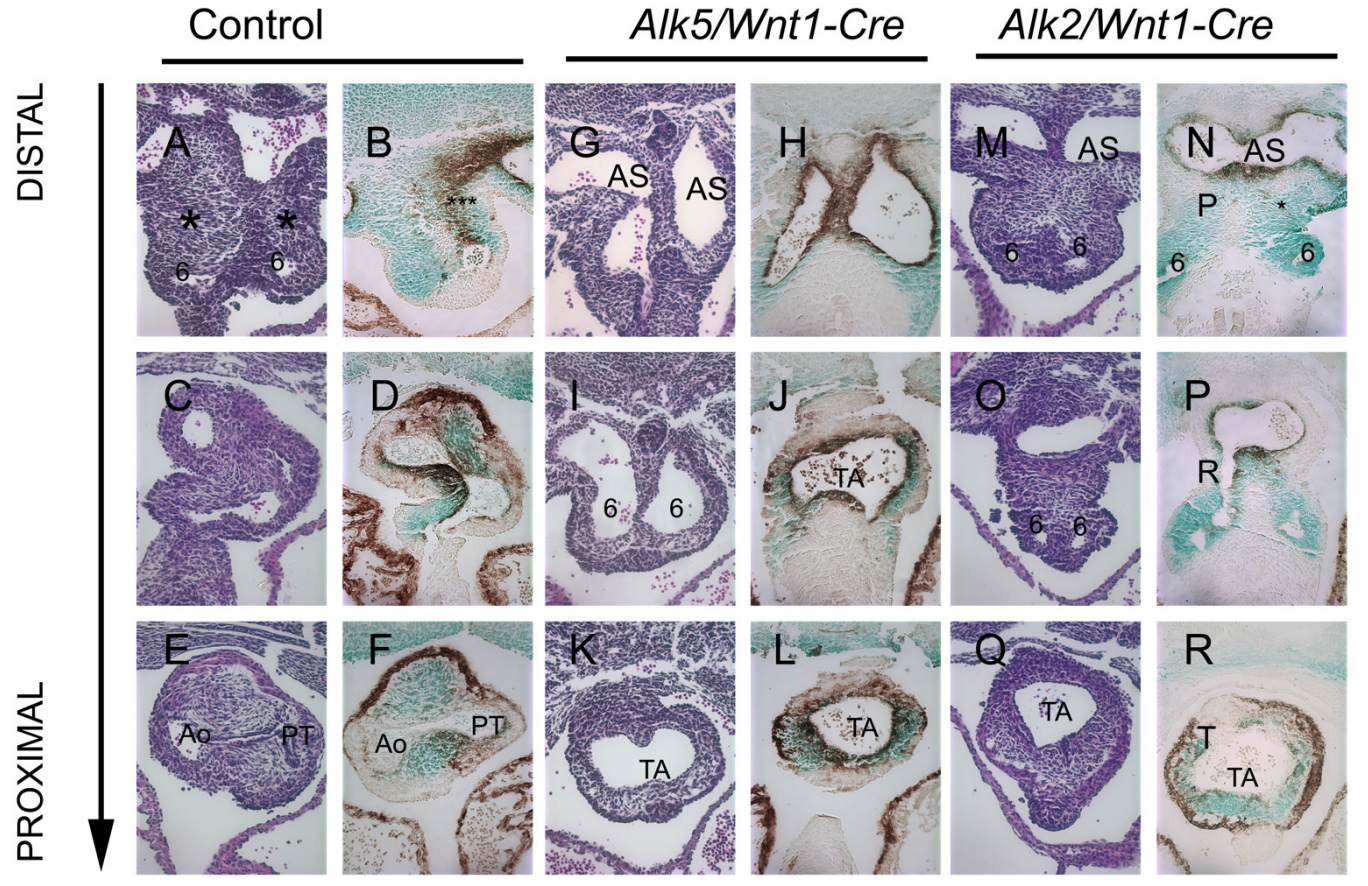
**Signaling via ALK5 and ALK2 controls different aspects of aortico-pulmonary septation**. Frontal sections from distal (top row) to proximal (bottom row) of the control (A-F), *Alk5/Wnt1-Cre *mutant (G-L) and *Alk2/Wnt1-Cre *mutant (M-R) samples (E11.5). A, C, E, G, I, K, M, O, Q, H&E staining; B, D, F, H, J, L, N, P, R, double staining for αSMA (brown) and β-galactosidase (green; R26R reporter assay). 6, the 6^th ^PAA; AS, aortic sac; TA, truncus arteriosus; Ao, Aorta; PT, pulmonary trunk; Asterisks in A, B, M and N depict the AP septal mesenchyme.

Septation of the outflow tract lumen begins in a cranial-to-caudal direction, starting distally in the aortic sac and proceeding toward the heart [24]. Initially, the condensed mesenchyme derived from the NC forms in the base of the aortic sac between the origins of 4^th ^and 6^th ^PAAs. Subsequently, two prongs of the developing aortico-pulmonary (AP) septum extend into the truncal cushions and the aortico-pulmonary septation complex crosses the aortic sac cranially. In ink-injected control embryos at E11.5, a characteristic conotruncal transition separating the truncus and conus could be seen as a twisted configuration, resulting from a change in orientation of the truncal and conal cushions (Fig. 5). In contrast, in *Alk5/Wnt1-Cre *mutants the outflow tract appeared unusually straight, failing to demonstrate the distinct conotruncal transition (Fig. 5B,D). This assay also clearly showed a dramatic reduction in the size of the aortic sac. Histological analysis of control samples displayed the characteristic rotation of the aortic sac and truncal OFT at the level where the AP septation takes place and verified the presence of the distinctive condensed AP-septal mesenchyme, which gradually divided the OFT to the aorta and the pulmonary trunk (asterisks in Fig. 6A). *R26R *lineage tracing showed that this tissue is derived from the NC, while immunostaining for α-SMA showed differentiation into smooth muscle (Fig 6B). In *Alk5/Wnt1-Cre *mutants the characteristic rotation of the aortic sac and truncal OFT fails to take place (Fig. 6G–L), and a properly formed AP-septum was not detectable (Fig. 6G,H). R26R lineage tracing demonstrated that the defects were not due to failure of NCCs to reach the OFT region. NC-derived cells around the abnomally bifurcated aortic sac, the abnormally large sixth PAAs and the truncus demonstrated strong αSMA staining (Fig. 6H,J,L). Recently, we showed that the NC-specific mutants of the related type I receptor, *Alk2*, display PTA as well [12]. In *Alk2/Wnt1-Cre *mutants, the rotation of the aortic sac and truncal OFT failed to occur (Fig. 6M–R) as seen in *Alk5/Wnt1-Cre *mutants. However, in *Alk2 *mutants the 6^th ^pair of the PAAs was grossly hypoplastic, and while the *Alk2/Wnt1-Cre *mutants displayed a noticeable amount of septal tissue between the 4^th ^and 6^th ^PAAs (Fig. 6M,N), the condensed septal mesenchyme lacking *Alk2 *failed to extend the prongs into the truncal cushions and to form the AP septum. Concurrently, the 6^th ^PAAs were losing their patency, which may have further contributed to the failed AP septation (Fig. 6M,O,Q). While CNCCs managed to migrate to the aortic sac and the truncal cushion level (Fig. 6N,P,R), immunostaining for αSMA appeared much weaker when compared to controls and *Alk5 *mutants, implying that ALK2-mediated signaling is involved in smooth muscle cell differentiation as previously suggested [12]. To conclude, while both *Alk2 *and *Alk5 *mutants demonstrate a failure in both the rotation of the aortic sac and the truncal OFT, and in the formation of the AP septum, the pathogenetic mechanisms behind these defects appear remarkably different.

### *Alk5*/*Wnt1-Cre *mutants display increased apoptosis of post-migratory neural crest cells

**Figure 7:**
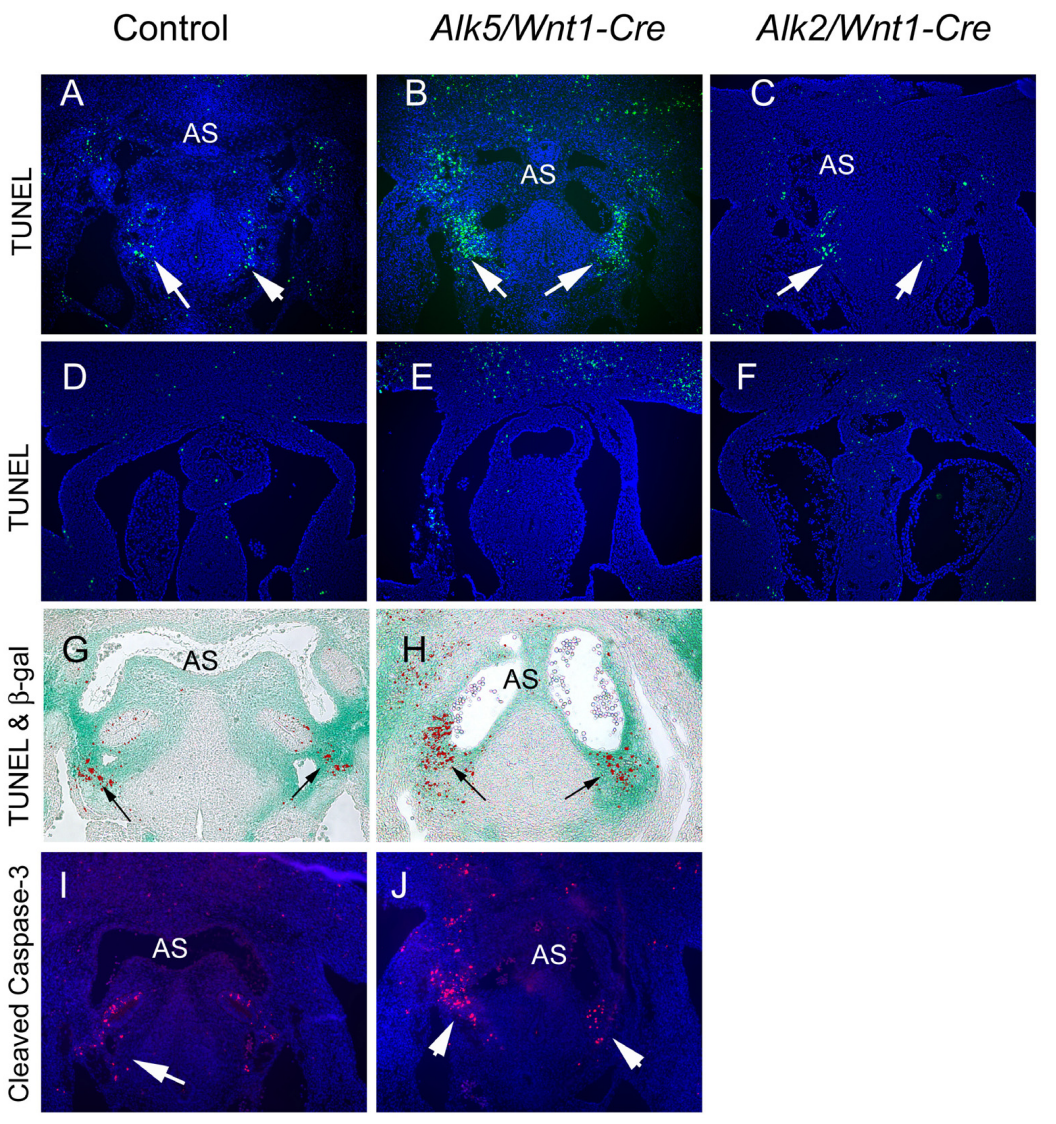
**Aberrant apoptosis of NCCs in *Alk5/Wnt1-Cre *mutants**. TUNEL (A-H) and Cleaved Caspase-3 (I, J) staining at E11.0 demonstrates a notable increase in apoptosis in *Alk5/Wnt1-Cre *mutants (B, H, J) on the aortic sac level when compared to controls (A, G, I) or *Alk2/Wnt1-Cre *mutants (C) (frontal sections), while sections on the OFT level do not demonstrate differences between controls (D) and *Alk5 *(E) or *Alk2 *(F) mutants. G,H, TUNEL staining of lacZ-stained embryos demonstrates that apoptotic cells are of neural crest origin. G, control; H, mutant. AS, aortic sac, arrows point to clusters of apoptotic cells surrounding the aortic sac.

As described above, *Alk5/Wnt1-Cre *mutants displayed an inadequate amount of AP-septal tissue in the base of the aortic sac between the origins of 4^th ^and 6^th ^PAAs. To analyze whether this phenotype resulted either from defective CNCC proliferation or inappropriate apoptosis, we used BrdU and TUNEL staining, respectively. While CNCC proliferation was not affected in *Alk5 *mutants (data not shown), we could detect a dramatic increase in the number of TUNEL positive cells in tissues surrounding the aortic sac including the site where the AP-septum forms (Fig. 7A–C). Dual staining for *lacZ *and TUNEL positive cells demonstrated that these cells were postmigratory CNCCs; this phenotype was already clearly detectable at E10.5. These results were confirmed by using immunostaining for cleaved caspase-3, another marker for apoptosis (Fig. 7I,J). In the chick, apoptotic neural crest-derived cells have also been found at the sites, where the prongs of the AP septum penetrate into the OFT cushion mesenchyme [25,26]. Thus, we compared apoptosis patterns also on the more proximal level, but found no detectable differences at E11.0 between *Alk5/Wnt1-Cre *mutants and controls (Fig. 7D,E). Unlike in *Alk5/Wnt1-Cre *mutant embryos, increased apoptosis of NC-derived cells is not responsible for the observed defects in the OFT septation in corresponding *Alk2 *mutants (Fig. 7C,E).

To conclude, our results suggest that in *Alk5/Wnt1-Cre *mutants a noticeable increase in apoptosis coincides with the abnormal patterning of the PAAs and the aortic sac, and with the failed AP-septation. These data support a specific role for ALK5 signaling, either directly or indirectly, in CNCC survival, since a similar apoptosis of NC-derived cells is not seen in *Tgfbr2/Wnt1-Cre *mutants [8,9].

### Pharyngeal organs fail to migrate in *Alk5*/*Wnt1-Cre *mutants

**Figure 8:**
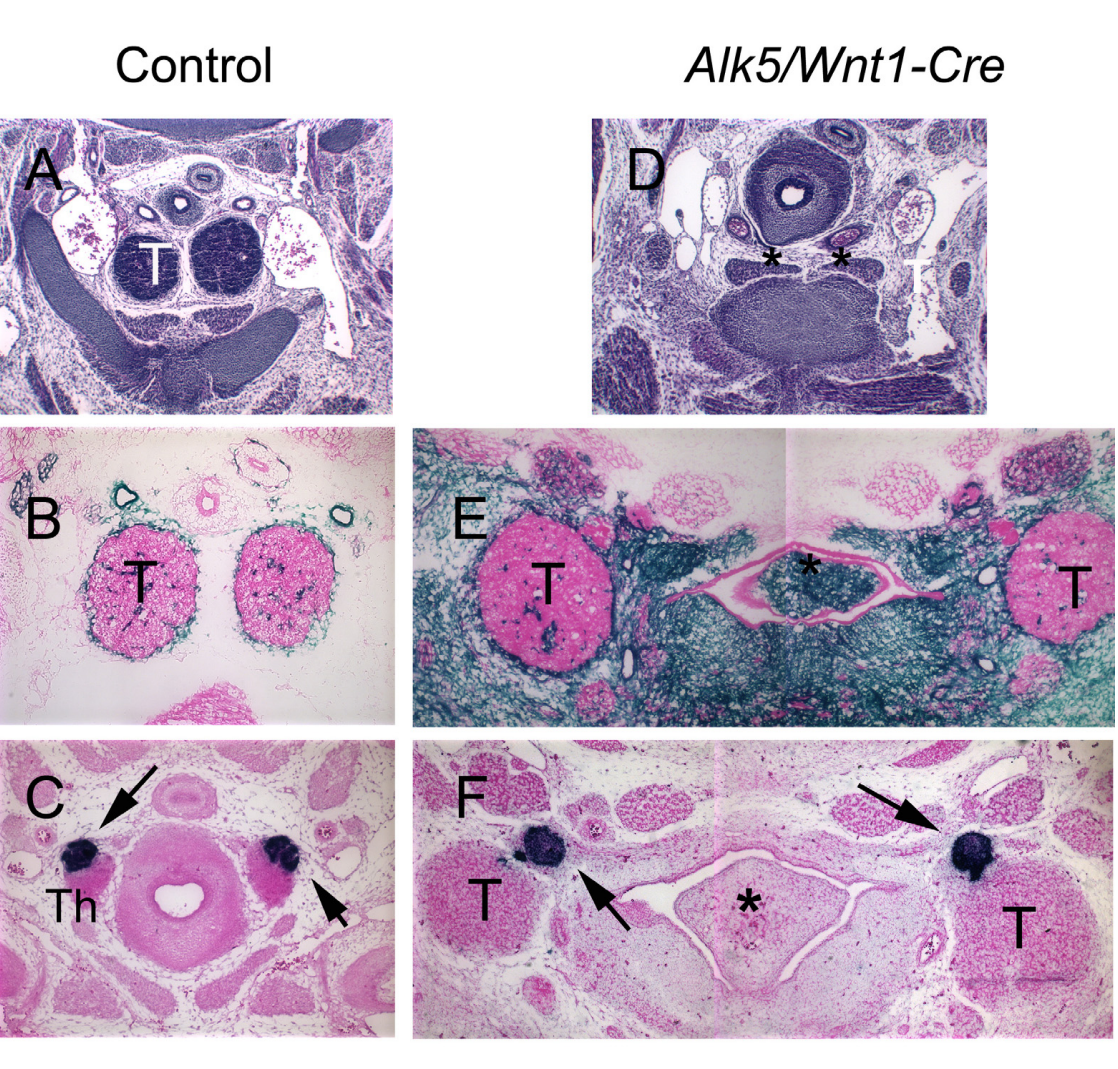
**Pharyngeal organs fail to migrate in *Alk5/Wnt1-Cre *mutants**. At E14.0, the thymus was not detectable in the superior mediastinum (asterisks) in *Alk5 *mutants (D), when compared to controls (A). Serial sectioning revealed that the thymic primordia had failed to descend and were seen bilaterally in the upper pharyngeal region (E, F) surrounded by neural crest derived mesenchyme (blue staining cells in E). In controls, the parathyroid glands were properly associated with the thyroid glands (arrows in C), while in *Alk5 *mutants the parathyroid glands were associated with the thymic primordia (arrows in F). However, both controls and mutants expressed parathyroid hormone (PTH) at comparable levels (blue staining in C and F). A and D, hematoxyllin and eosin staining; B and E, R26 R lineage tracing assay – counterstaining with eosin; C and F, section in situ hybridization for PTH – counterstaining with eosin. T, thymus; Th, thyroid; asterisks in D depict the absence of the thymic primordia; asterisks in E and F depict the tongue.

In addition to the cardiac OFT, development of pharyngeal organs, i.e., the parathyroid glands and the thymus was also abnormal in *Alk5/Wnt1-Cre *mutants (see Figs. 1 and 8). Normally the thymus develops from the third pharyngeal pouch endoderm and migrates caudally to its final location in the superior mediastinum as seen in controls at E14 (Fig. 8A,B). In contrast, the thymic primordia of the *Alk5 *mutant littermates failed to descend caudally, and were located bilaterally in the neck region, where they were surrounded by neural crest-derived mesenchyme (Fig. 8D,E). The fate determination assay demonstrated that the thymic primordia were equally populated by NCCs both in controls and *Alk5 *mutants (Fig. 8B,E). Likewise the parathyroid glands failed to migrate normally in *Alk5/Wnt1-Cre *mutants. During normal development, the parathyroids first migrate in association with the thymic primordia, until they reach the thyroids in the neck region as seen in Fig 8C. In *Alk5/Wnt1-Cre *mutants, the parathyroids remained associated with the thymic primordia, and despite this abnormal rostral location, expression of parathyroid hormone was indistinguishable between *Alk5 *mutants and controls at E14 (Fig. 8C,F). To conclude, the observed pharyngeal organ phenotypes were also in striking contrast to those seen in *Tgfbr2/Wnt1-Cre *mutants [8,9].

## Discussion

During the last few years the *Wnt1-Cre *transgenic driver line has proven to be a powerful tool for tissue-specific gene deletion in NCCs [12,13,27,28]. Using this approach, several studies have independently shown that the NC-specific deletion of the *Tgfbr2 *gene leads to a distinct set of calvarial, facial and cardiac defects [8,9,20,29]. Interestingly, these defects appear quite different both in the craniofacial and pharyngeal regions, including the heart, when compared to the corresponding mutants of *Alk5*, which encodes the TGF-β type I receptor, a prototypical binding partner of TGF-βRII [19] and the present study). While *Tgfbr2/Wnt1-Cre *mutants as well as mice deficient in *Tgf-β2 *display the PTA type A4 (truncus arteriosus with interrupted aortic arch [30]), *Alk5/Wnt1-Cre *mutants reported here demonstrate earlier patterning defects of the PAAs, which is particularly obvious in the 3^rd ^pair of the PAAs. Moreover, the *Alk5/Wnt1-Cre *mutants display an abnormal patterning of the aortic sac and defective AP septation leading to PTA, reminiscent of type A2 (= truncus artriosus with no main pulmonary artery segment present [30]). However, our results also demonstrate that significant hypoplasia of the aortic sac leads to a severe shortening of the ascending truncal arch, which masks possible defects in derivatives of the 4^th ^PAAs, i.e., interruption of the aortic arch. These observed differences are likely due to substantial apoptosis of *Alk5-*deficient post-migratory neural crest cells, which is clearly detectable at E10.5, whereas similar intense cell death has not been reported in *Tgfbr2/Wnt1-Cre *mutants[9].

Our present results suggest that while TGF-β signaling in cardiac NCCs is predominantly mediated via the ALK5/TGF-βRII receptor complex, ALK5 also mediates signaling of other related ligands, which are either directly or indirectly required for appropriate NCC survival. In fact, it has been shown that, besides TGF-βRII, ALK5 can also form a complex with the Activin type IIB receptor to activate downstream Smads 2/3 [18,31]. Furthermore, a subset of TGF-β-related growth and differentiation factors (GDFs), e.g., GDF8, GDF9, GDF11 and GDF15 could induce these events [17,18,32,33]. Although relevant *Gdfs 8, 9 11 *and *15 *are not expressed in the developing heart, nor do the mice deficient in these *Gdfs *display developmental cardiac defects, we cannot exclude the possibility that circulating GDFs, perhaps in concert with TGF-βs may contribute to NCC survival during cardiac and pharyngeal morphogenesis.

We specifically studied apoptosis at the level of the aortic sac, where the AP-septum forms between the origins of 4^th ^and 6^th ^PAAs. Already at E10.5, we could see intense apoptosis among the postmigratory NCCs in the mesenchyme surrounding the aortic sac at the site where the prospective AP septum forms, i.e., this cell death precedes the AP septal defect seen in mutants. Although some NC-derived cells appeared to be differentiating to smooth muscle cells in the OFT (Fig. 6), we could never detect the AP septum forming in Alk5/Wnt1-Cre mutants between E10.5 and E11.5. These findings suggest that the pool of cells forming the AP septum is severely affected by the cell death. Moreover, it is likely that these cells forming the AP-septum die the before majority of them can differentiate to smooth muscle cells.

Several *in vitro *studies have suggested an indispensable role for TGF-β-signaling in differentiation of NCCs into smooth muscle cells. Moreover, a recent *in vivo *study suggested that mice lacking *Tgfbr2 *in CNCCs display defective NCC differentiation into αSMA-positive cells in the AP septum [9], although this result was later disputed by another study [8]. Our immunohistochemical staining of αSMA in the OFT unequivocally demonstrated that signaling via ALK5 is not required for smooth muscle differentiation *in vivo*. Moreover, it has been suggested that deletion of *Tgfbr2 *in NCCs leads to other phenotypic features reminiscent of those seen in the velocardiofacial/DiGeorge syndrome (VCF/DGS) [9] caused by a deletion of the so called DiGeorge critical region (*DGCR*) on chromosome 22q11 [34,35]. Our present results suggest that although many of the observed phenotypes seen in *Alk5/Wnt1-Cre *mutants superficially resemble those seen in VCF/DGS, a detailed examination shows that the NC-specific abrogation of *Alk5 *does not lead to VCF/DGS-like phenotypes. Firstly, while the pharyngeal organ migration fails in *Alk5/Wnt1-Cre *mutants, perhaps as a result of increased mesenchymal cell death in the pharyngeal region, both the thymus, thyroid and parathyroid seem to develop relatively normally on the histological level in these mutants. Secondly, the NCC death seen in *Alk5 *mutants affects a predominantly postmigratory population of NCCs, while genes located in the DGCR, i.e., *Tbx1 *and *CrkL*, control NCC survival earlier at E8.5-E10 by regulating proliferation of the secondary heart field (SHF), and endoderm expansion, which in turn provides survival signal for NCCs allowing them to populate the pharyngeal region [36-39].

NCC ablation in the chick has been shown to lead to PTA and to a failure of addition of myocardium from the secondary heart field [40]. It was suggested that the defective migration of cells from the secondary heart field to the OFT in turn resulted in shortening and inappropriate rotation of the OFT, leading to mal-alignment of the arterial pole with the ventricles [41]. While the detected OFT phenotype in *Alk5/Wnt1-Cre *mutants shared many similarities with that seen in the chick NC ablation models, e.g., PTA and the hypoplastic aortic sac, our current results suggest that the secondary heart field is not severely affected in *Alk5 *mutants (data not shown). Since we could not detect appropriate rotation of the OFT in neural crest-specific mutants of *Alk5 *or *Alk2*, it appears that cells derived from the NC, as well as those from the SHF, are mutually required for proper OFT rotation in mice. However, it appears that these two TGF-β/BMP type I receptors contribute to OFT rotation through different mechanisms. In Alk5 mutants there is very little, if any, detectable septal mesenchyme present, and thus it could be argued that in these mutants OFT rotation fails due to a lack of penetration of septal prongs into the cushion mesenchyme. In contrast, in *Alk2/Wnt1*-*Cre *mutants a sizeable septal mesenchyme could be seen, still without any obvious looping of the aortic sac and truncal OFT, suggesting that the mere presence of the septal mesenchyme, without correct smooth muscle cell differentiation, is not sufficient for OFT rotation.

## Conclusion

In this study, we have deleted the TGF-β type I receptor (*Alk5*) gene specifically in the mouse neural crest (NC) cell lineage. Our data suggest that ALK5 is required cell autonomously in the NC to mediate non-redundant signaling events that are essential for appropriate patterning of the pharyngeal organs and cardiac OFT. The cardiac and pharyngeal defects observed in the NC-specific *Alk5 *mutants differ significantly from those seen in corresponding mutants lacking the TGF-β type II receptor, suggesting that signaling mediated by ALK5 is not limited to the classical TGF-β ligands during cardiac/pharyngeal development.

## Methods

### *Alk5*/*Wnt1Cre *mice

*Alk5 (*and *Alk2*) mutant and control embryos were generated by mating Alk5^ko/+^(Alk2^ko/+^)/Wnt1-Cre male mice with females homozygous for the Alk5^flox ^(Alk2^flox^) allele and the R26R reporter[12,19]. Genotyping was performed by PCR as described earlier [21,42]. *Wnt1-Cre *mice were kindly provided by A. McMahon (Harvard University) and R26R reporter mice were obtained from the Jackson laboratories. All studies were carried out at the Animal Care Facility of the Saban Research Institute of Childrens Hospital Los Angeles in accordance with institutional guidelines.

### Timed mataings

Mice were mated during the dark period of the controlled light cycle; presence of vaginal plugs was designated as day 0 hour 0. Females were euthanized by CO_2_, and embryos were collected in Hanks' balanced salt solution on ice.

### Histological analyses

Embryos (E17) were fixed with 4% paraformaldehyhe for 14 hours, dehydrated and embedded in paraffin wax. Sections (7–8 um) were stained with Hematoxylin and Eosin (n≥3 for each genotype). For lineage tracing analyses, embryos were stained for β-galactosidase activity as described [43]. Briefly, the specimens (E11.0 – E11.5) were fixed in 4% paraformaldehyde for 30 minutes at room temperature, washed 3 times for 10 minutes in the detergent wash and developed for 2–12 hours in X-gal solution (n≥3 for each genotype). For immunohistochemistry, fixed sections from tissues harvested at E10.5 – E11.5 were stained with monoclonal α-smooth muscle actin (DAKO), cleaved caspase-3 (Cell Signaling) or phophohistone-H3 (Cell Signaling) antibodies. TUNEL assays were performed using the DeadEnd fluorometric TUNEL system (Promega). In each assay 3 or more embryos were analyzed for each genotype.

### Ink and casting dye injections

Embryos (E10.0 – E13.0) were dissected and placed in ice cold PBS (n≥3 per genotype in each time point). Using a pulled glass pipette, India ink or Yellow casting dye (Connecticut Valley Biological Supply) was injected into the ventricles until ink/dye penetrated small vessels. Embryos were postfixed in 10% buffered formalin for 12 hours, dehydrated and cleared in benzylbenzoate: benzyl alcohol (2:1v/v).

### Expression analyses

To visualize parathyroid hormone expression we used in situ hybridization and an antisense probe corresponding to nucleotides 97–534 (kindly provided by Nancy Manley) as described [44].

## Authors' contributions

J.W. did most of the mouse dissections and analyses, A.N. did some of the histological analyses, J.L. generated the *Alk5*^*FXFX*^mice, M.D. did some of the expression analyses, H.M.S. participated in design and provided the *Tgfbr2 *mutant embryos and V.K. generated the *Alk2*^*FXFX *^mice, designed and supervised the experiments and wrote the manuscript. All authors have read and approved the final manuscript.
